# Editorial: Immunometabolic Regulations in Adaptive and Innate Immune Cells Shapes and Re-Directs Host Immunity

**DOI:** 10.3389/fimmu.2017.00852

**Published:** 2017-07-24

**Authors:** Ping-Chih Ho, Christoph Hess

**Affiliations:** ^1^Department of Fundamental Oncology, University of Lausanne, Lausanne, Switzerland; ^2^Ludwig Center for Cancer Research, University of Lausanne, Lausanne, Switzerland; ^3^Department of Biomedicine, Immunobiology, University of Basel, Basel, Switzerland

**Keywords:** immunometabolism, metabolic reprogramming, tumor microenvironment, autoimmunity, immune suppression

**Editorial on the Research Topic**

**Immunometabolic Regulations in Adaptive and Innate Immune Cells Shapes and Re-Directs Host Immunity**

## Metabolism

Metabolism, a nightmare for many students and, let’s be honest, not-anymore students alike. Complicated pathways and crosstalks between metabolites and metabolic enzymes—with little tangible relation to any real-life biology. In the past two decades, however, very concrete clinical scenarios, such as cancer, diabetes, and immunity, have become inseparably tied to cellular metabolic dysregulation. With unprecedented breath and depth new technologies, such as metabolomics, proteomics, RNA-sequencing, and epigenomics, allowed interrogating cellular metabolism, its links to organismal metabolic changes, as well as its regulation and biologic importance in health and disease.

In this research topic, which entails 11 invited review articles, we are focusing on how metabolic regulation fine-tunes activation, differentiation and acquisition of effector function among both innate and adaptive immune cells.

Starting out, a review by Wei et al. provides an overview on how regulatory circuits of oxygen- and nutrient-sensing machineries orchestrate T cell responses. The authors further discuss the role of autophagy and the redox- and NAD+/NADH balance in T cell differentiation and activation. A review article by Gardiner and Finlay discusses the uniqueness of metabolic regulation in guiding NK cell activation and NK cell dependent immune responses. The work by Langston et al. covers, in detail, mechanisms by which distinct signals induce a metabolic shift in macrophages, and how such metabolic adaptation sustains signaling pathways to drive macrophage polarization. The authors further discuss how metabolic regulation in macrophages centers on mitochondria to enforce cell activation. The review by Sancho et al. discusses how mitochondrial activity, and in particular the dynamics of the electron transport chain, acts as a rheostat to modulate signaling, transcription, and the epigenome of innate immune cells. Chao et al. go on to discuss how mitochondria regulate T cell activation, differentiation, and function. The authors of this paper further highlight how mitochondrial fitness and dynamics relate to T cell immunity in cancer, infection, autoimmunity, and during aging.

The review by Binger et al. is focused on metabolic reprogramming in CD4^+^ helper T cells, with a particular emphasis on Th17 and regulatory T cells (Tregs). The article highlights the metabolic demands of Th17 cells, and describes how metabolic regulation in Th17 cells contributes to autoimmunity. Kolev and Kemper then discuss the role of complement, an emerging player of immunometabolic regulation. The authors postulate that this ancient arm of the immune system may indeed have coevolved with immunometabolism.

The last four papers of this research topic emphasize contributions of immunometabolic regulation in cancer, atherosclerosis, and obesity. First, Renner et al. discuss metabolic communication and competition between immune- and cancer-cells in the tumor microenvironment and propose therapeutic strategies to enhance anti-tumor immunity by augmenting metabolic fitness of tumor-infiltrating immune cells. Patsoukis et al. highlight how co-inhibitory receptors tailor metabolic reprogramming and pathway usage in T cell subsets. Since tumor-infiltrating T cells often express co-inhibitory receptors, understanding the metabolic circuits controlled by these receptors may provide a springboard toward reinvigorating cancer-directed T cell immune responses *via* metabolic reprogramming. Based on the idea that tumor-infiltrating T cells may face a metabolic crisis in the tumor microenvironment, Irving et al. discuss how metabolic rewiring may be an attractive strategy for designing new T cell-based cancer immunotherapies, particularly chimeric antigen receptor T cell therapies. In addition to T cells, Geeraerts et al. describe how the metabolic environment in tumors and in atheromatous lesions affects macrophage polarization and function *via* intervening with cellular metabolic processes. This article further discusses how altered systemic metabolism among obese individuals, and mice, may promote metabolic disorders, including insulin resistance, by modulating inflammatory properties of adipose tissue macrophages.

In summary, this research topic highlights the tremendous progress that has been made in understanding how metabolic reprogramming underpins immune cell differentiation, activation, and effector function. However, and as also highlighted by the various contributions, key questions regarding the detailed molecular mechanisms by which metabolic pathways control immune cell responses are still unresolved. Specifically, important metabolic checkpoints remain to be described, and it is still an enigma how metabolic checkpoints crosstalk to orchestrate immune responses. We hope that this research topic not only will serve as a valuable resource but also that it will also provoke questions and help formulating relevant next-generation research questions—with the ultimate goal to exploit immunometabolic regulation to the benefit of patients.

## Author Contributions

All authors listed have made a substantial, direct, and intellectual contribution to the work and approved it for publication.

## Conflict of Interest Statement

These authors declare no financial relationship that can cause conflict of interest with the contents of this work.

